# Supplementary material for: Modeling glioblastoma heterogeneity as a dynamic network of cell states
Source: Mol Syst Biol. 2021 Sep 16;17(9):e10105. doi: 10.15252/msb.202010105 (PMC8444284; doi:10.15252/msb.202010105)
Supplement: Supplementary file 6 — Source Data for Figure 5 [file MSB-17-e10105-s004.zip › Figure5A_sourcedata/GSEA_3071/hallmarks_stateI.GseaPreranked.1623742531165/HALLMARK_APICAL_JUNCTION.html]

Details for gene set HALLMARK\_APICAL\_JUNCTION[GSEA]

|  || Dataset | state13071 |
| Phenotype | NoPhenotypeAvailable |
| Upregulated in class | na\_pos |
| GeneSet | HALLMARK\_APICAL\_JUNCTION |
| Enrichment Score (ES) | 0.32247895 |
| Normalized Enrichment Score (NES) | 1.7318839 |
| Nominal p-value | 0.0062305294 |
| FDR q-value | 0.019350294 |
| FWER p-Value | 0.167 |
Table: GSEA Results Summary

  

Fig 1: Enrichment plot: HALLMARK\_APICAL\_JUNCTION      
 Profile of the Running ES Score & Positions of GeneSet Members on the Rank Ordered List

  

| PROBE | GENE SYMBOL | GENE\_TITLE | RANK IN GENE LIST | RANK METRIC SCORE | RUNNING ES | CORE ENRICHMENT || 1 | TGFBI |  |  | 17 | 1.674 | 0.0394 | Yes |
| 2 | CDH11 |  |  | 59 | 1.296 | 0.0618 | Yes |
| 3 | ITGA10 |  |  | 70 | 1.217 | 0.0911 | Yes |
| 4 | THY1 |  |  | 94 | 1.114 | 0.1139 | Yes |
| 5 | FBN1 |  |  | 138 | 0.944 | 0.1264 | Yes |
| 6 | VCL |  |  | 162 | 0.891 | 0.1433 | Yes |
| 7 | EGFR |  |  | 175 | 0.864 | 0.1627 | Yes |
| 8 | ITGA2 |  |  | 194 | 0.839 | 0.1797 | Yes |
| 9 | VCAN |  |  | 197 | 0.833 | 0.2012 | Yes |
| 10 | MMP2 |  |  | 284 | 0.697 | 0.1947 | Yes |
| 11 | ITGA3 |  |  | 297 | 0.677 | 0.2091 | Yes |
| 12 | ACTG2 |  |  | 313 | 0.659 | 0.2222 | Yes |
| 13 | BMP1 |  |  | 346 | 0.631 | 0.2296 | Yes |
| 14 | SHC1 |  |  | 361 | 0.624 | 0.2421 | Yes |
| 15 | MPZL1 |  |  | 363 | 0.624 | 0.2583 | Yes |
| 16 | ITGB1 |  |  | 371 | 0.615 | 0.2725 | Yes |
| 17 | AMIGO2 |  |  | 377 | 0.610 | 0.2872 | Yes |
| 18 | MYL9 |  |  | 417 | 0.576 | 0.2912 | Yes |
| 19 | SDC3 |  |  | 471 | 0.534 | 0.2899 | Yes |
| 20 | SKAP2 |  |  | 483 | 0.526 | 0.3006 | Yes |
| 21 | TJP1 |  |  | 557 | 0.477 | 0.2921 | Yes |
| 22 | CD276 |  |  | 572 | 0.469 | 0.3004 | Yes |
| 23 | VCAM1 |  |  | 603 | 0.450 | 0.3036 | Yes |
| 24 | NF1 |  |  | 617 | 0.445 | 0.3116 | Yes |
| 25 | CD99 |  |  | 659 | 0.430 | 0.3111 | Yes |
| 26 | RSU1 |  |  | 660 | 0.430 | 0.3225 | Yes |
| 27 | MYH9 |  |  | 701 | 0.411 | 0.3217 | No |
| 28 | CX3CL1 |  |  | 828 | 0.373 | 0.2950 | No |
| 29 | NECTIN2 |  |  | 845 | 0.366 | 0.3001 | No |
| 30 | ADAMTS5 |  |  | 876 | 0.357 | 0.3008 | No |
| 31 | SGCE |  |  | 891 | 0.353 | 0.3061 | No |
| 32 | RASA1 |  |  | 935 | 0.342 | 0.3027 | No |
| 33 | ITGB4 |  |  | 1125 | 0.302 | 0.2558 | No |
| 34 | RRAS |  |  | 1160 | 0.297 | 0.2538 | No |
| 35 | EPB41L2 |  |  | 1170 | 0.295 | 0.2590 | No |
| 36 | PARVA |  |  | 1301 | 0.268 | 0.2284 | No |
| 37 | CTNND1 |  |  | 1338 | 0.261 | 0.2248 | No |
| 38 | ACTN1 |  |  | 1364 | 0.258 | 0.2244 | No |
| 39 | MSN |  |  | 1379 | 0.255 | 0.2271 | No |
| 40 | MYH10 |  |  | 1670 | -0.276 | 0.1502 | No |
| 41 | PIK3R3 |  |  | 1797 | -0.293 | 0.1214 | No |
| 42 | KCNH2 |  |  | 1937 | -0.311 | 0.0893 | No |
| 43 | CD274 |  |  | 1966 | -0.314 | 0.0895 | No |
| 44 | ADAM9 |  |  | 2063 | -0.328 | 0.0703 | No |
| 45 | SHROOM2 |  |  | 2230 | -0.360 | 0.0317 | No |
| 46 | HRAS |  |  | 2276 | -0.367 | 0.0283 | No |
| 47 | PFN1 |  |  | 2305 | -0.373 | 0.0301 | No |
| 48 | PKD1 |  |  | 2410 | -0.394 | 0.0103 | No |
| 49 | SORBS3 |  |  | 2428 | -0.398 | 0.0159 | No |
| 50 | EVL |  |  | 2511 | -0.416 | 0.0031 | No |
| 51 | ADAM23 |  |  | 2557 | -0.428 | 0.0014 | No |
| 52 | SIRPA |  |  | 2562 | -0.431 | 0.0116 | No |
| 53 | FSCN1 |  |  | 2569 | -0.432 | 0.0213 | No |
| 54 | JAM3 |  |  | 2654 | -0.455 | 0.0090 | No |
| 55 | TUBG1 |  |  | 2715 | -0.475 | 0.0041 | No |
| 56 | YWHAH |  |  | 2725 | -0.479 | 0.0142 | No |
| 57 | CADM3 |  |  | 2745 | -0.484 | 0.0215 | No |
| 58 | ARHGEF6 |  |  | 2799 | -0.501 | 0.0194 | No |
| 59 | LAMB3 |  |  | 2874 | -0.529 | 0.0119 | No |
| 60 | GNAI2 |  |  | 2889 | -0.535 | 0.0220 | No |
| 61 | NF2 |  |  | 2972 | -0.583 | 0.0136 | No |
| 62 | DLG1 |  |  | 3043 | -0.628 | 0.0100 | No |
| 63 | GAMT |  |  | 3141 | -0.723 | 0.0009 | No |
| 64 | CDH4 |  |  | 3161 | -0.742 | 0.0151 | No |
| 65 | MDK |  |  | 3224 | -0.811 | 0.0185 | No |
| 66 | NRXN2 |  |  | 3361 | -1.072 | 0.0074 | No |
| 67 | LIMA1 |  |  | 3429 | -1.353 | 0.0238 | No |
Table: GSEA details [plain text format]

  

Fig 2: HALLMARK\_APICAL\_JUNCTION: Random ES distribution      
 Gene set null distribution of ES for **HALLMARK\_APICAL\_JUNCTION**
